# The *Drosophila* Post-mating Response: Gene Expression and Behavioral Changes Reveal Perdurance and Variation in Cross-Tissue Interactions

**DOI:** 10.1534/g3.119.400963

**Published:** 2020-01-06

**Authors:** Nicole R. Newell, Surjyendu Ray, Justin E. Dalton, Julia C. Fortier, Joyce Y. Kao, Peter L. Chang, Sergey V. Nuzhdin, Michelle N. Arbeitman

**Affiliations:** * Department of Biomedical Sciences, College of Medicine, Florida State University, Tallahassee, FL and; † Department of Biological Sciences, University of Southern California, Los Angeles, CA

**Keywords:** cross-tissue interactions, Drosophila, gene expression, GWAS, post-mating, RNA-seq, sleep, Genetics of Sex

## Abstract

Examining cross-tissue interactions is important for understanding physiology and homeostasis. In animals, the female gonad produces signaling molecules that act distally. We examine gene expression in *Drosophila melanogaster* female head tissues in 1) virgins without a germline compared to virgins with a germline, 2) post-mated females with and without a germline compared to virgins, and 3) post-mated females mated to males with and without a germline compared to virgins. In virgins, the absence of a female germline results in expression changes in genes with known roles in nutrient homeostasis. At one- and three-day(s) post-mating, genes that change expression are enriched with those that function in metabolic pathways, in all conditions. We systematically examine female post-mating impacts on sleep, food preference and re-mating, in the strains and time points used for gene expression analyses and compare to published studies. We show that post-mating, gene expression changes vary by strain, prompting us to examine variation in female re-mating. We perform a genome-wide association study that identifies several DNA polymorphisms, including four in/near Wnt signaling pathway genes. Together, these data reveal how gene expression and behavior in females are influenced by cross-tissue interactions, by examining the impact of mating, fertility, and genotype.

In animals, organs and tissues communicate through secreted signaling molecules to coordinate physiological functions. For example, interactions between the brain and reproductive organs in mammals, via signaling molecules in the hypothalamus-pituitary-gonadal axis, are responsible for the coordination of reproduction, metabolism, and behavior (reviewed in Meethal and Atwood 2005; Della Torre *et al.* 2014). Organs communicate to maintain homeostasis, so understanding how perturbation of one organ alters gene expression and functions of other organs is an important goal for understanding and treating human disease (reviewed in Schadt 2009). The fruit fly, *Drosophila melanogaster*, has complex organ systems with cross-tissue interactions mediated by genes that are conserved across phyla. Thus, *Drosophila* is a tractable *in vivo* model system to study cross-tissue interactions, with a range of investigations on cross-tissue and cross-organ interactions already performed (for example see Hudry *et al.* 2019; Scopelliti *et al.* 2019; and reviewed in Rajan and Perrimon 2011; Droujinine and Perrimon 2013; Droujinine and Perrimon 2016; Jayakumar and Hasan 2018; Ahmad *et al.* 2019). In this study, we analyze cross-tissue interactions associated with female reproduction, with a focus on how these interactions impact gene expression in adult head tissues and behavior.

In *Drosophila*, signaling molecules are known to mediate cross-talk between the female nervous system, fat body (a tissue akin to the mammalian adipose and liver tissues), endocrine, gut, and reproductive tissues. These signals coordinately regulate aspects of physiology, energy homeostasis, immunity, and lifespan with reproduction (reviewed in Toivonen and Partridge 2009; Rajan and Perrimon 2011; Droujinine and Perrimon 2016; Ahmad *et al.* 2019). For example, *Drosophila* has eight insulin-like signaling peptides (Ilps1-8), and expression of Ilps 2, 3, and 5 in adult brain median neurosecretory cells regulates the rate of female germline cell division, through binding to insulin receptor (InR) on germline stem cells (Ikeya *et al.* 2002; Lafever and Drummond-Barbosa 2005; Hsu and Drummond-Barbosa 2009). Based on analysis of *InR* mutants, it is also clear that insulin signaling regulates the production of juvenile hormone (JH; Tu *et al.* 2005), a sesquiterpenoid produced in the corpus allatum (insect endocrine gland, Figure S1). JH stimulates production of 20-hydroxyecdysone (ecdysone), a steroid hormone produced in the ovaries, during adult stages (Figure S1, blue arrow; Tu *et al.* 2002; Tu *et al.* 2005). The production of ecdysone then stimulates production of yolk proteins in the gonadal fat body. Yolk proteins, an energy resource, are released into the hemolymph and absorbed by the ovaries (reviewed in Gruntenko and Rauschenbach 2008), to coordinate energy homeostasis and reproductive functions. The ecdysone signaling pathway is also essential for germline development and maintenance of germline stem cells (reviewed in Ables and Drummond-Barbosa 2017; Swevers 2019). Additionally, there is JH production post-mating that triggers remodeling of the midgut, resulting in a larger organ in anticipation of greater nutrient demands after mating (Reiff *et al.* 2015). Thus, the signaling pathways known to coordinate reproduction and physiology are complex, acting from and on distinct tissues and organs.

These signaling pathway interactions are also important for the female post-mating response (PMR), which includes increased egg laying (Chen *et al.* 1988) and feeding (Carvalho *et al.* 2006), a preference for both yeast and salt instead of carbohydrates (Ribeiro and Dickson 2010; Vargas *et al.* 2010; Walker *et al.* 2015), decreased intestinal transit (Cognigni *et al.* 2011; Apger-Mcglaughon and Wolfner 2013), decreased receptivity to mating (Manning 1962; Chen *et al.* 1988; Aigaki *et al.* 1991; Chapman *et al.* 2003), decreased daytime sleep (Isaac *et al.* 2010; Garbe *et al.* 2016; Dove *et al.* 2017) and lowered immune response (Fedorka *et al.* 2007; Short and Lazzaro 2010; Short *et al.* 2012). During copulation, peptides are transferred to the female in the male seminal fluid that induce the PMR (reviewed in Wolfner 1997; Wolfner 2002; Avila *et al.* 2011; Sirot *et al.* 2014). One critical peptide, sex-peptide (SP), which acts through a G-protein coupled receptor called sex-peptide receptor (SPR; Yapici *et al.* 2008), induces the short-term PMR (<1 day). The gradual release of SP bound to sperm is required for the long-term PMR (1-7 days, Peng *et al.* 2005). SP stimulates production of JH in the corpus allatum and ecdysone in the ovaries, with this SP-dependent increase of ecdysone driving the proliferation of germline stem cells (Figure S1, purple arrows; Moshitzky *et al.* 1996; Ameku and Niwa 2016). Further evidence that these signaling pathways mediate the PMR is that perturbation of the insulin signaling pathway, ecdysone, or JH impacts female reproductive behaviors (Ringo *et al.* 1991; Ringo *et al.* 2005; Wigby *et al.* 2011; Ganter *et al.* 2012; Watanabe and Sakai 2016).

Several genomic studies have determined the impact of mating on gene expression in female adult tissues (summary in Table S1). These include studies of whole flies at several time-points ≤ 24 hr post-mating (Lawniczak and Begun 2004; Mcgraw *et al.* 2004; Mcgraw *et al.* 2008; Short and Lazzaro 2013), studies of whole flies that examine the impact of single *vs.* double mating (Innocenti and Morrow 2009), and studies of adult flies with no gonadal tissues, examined immediately post-mating (Parisi *et al.* 2010). Tissue-specific gene expression studies include an analysis of abdominal and head/thorax tissues 3-6 hr post-mating (Gioti *et al.* 2012), reproductive tract tissues (minus the ovaries) at 0, 3, 6, and 24 hr post-mating (Mack *et al.* 2006), oviduct tissues at 3-hours post-mating (Kapelnikov *et al.* 2008), and head tissues at 0-2, 24, 48, and 72 hr post-mating (Dalton *et al.* 2010). A recent study compared gene expression changes in the head/thorax and abdomen 3-hours post-mating in both males and females (Fowler *et al.* 2019). There are additional population-level studies examining the effect of genetic background on gene expression changes post-mating (Fear *et al.* 2016; Delbare *et al.* 2017). It is clear from these studies that the PMR is tissue-specific, temporally dynamic, and influenced by genotype.

Here, using RNA-sequencing (RNA-seq), we examine gene expression changes in age-matched female adult head tissues (comparisons are shown in Table 1); these conditions/tissues have not been examined previously. Head tissue is predominantly comprised of nervous system and pericerebral fat body tissues, so we gain insight into expression changes that mediate behavior and metabolism. In this study, we use *tudor* (*tud*) mutants to generate males and females that lack germline tissues (Boswell and Mahowald 1985; and reviewed in Thomson and Lasko 2005). We compare gene expression in virgins with a germline to those lacking a germline, and show that the absence of the germline results in altered expression of genes with a known function in nutrient homeostasis. We also examine one- and three-day(s) post-mating gene expression changes compared to virgin controls. We compare gene expression in post-mated females (with and without a germline) to virgin controls, as well as gene expression in post-mated Berlin females that had been mated to males (with and without a germline) compared to virgin controls (Table 1). We find that in all conditions, the female post-mating response results in changes in expression of genes that function in metabolism, however, each comparison had largely different genes with expression changes. We perform gene set enrichment analysis and find that only one condition, three-day, post-mated females lacking a germline, has genes with expression changes that are enriched with several ‘neuronal’ and ‘behavioral’ biological process terms.

**Table 1:** Description of comparisons for gene expression analyses. The comparisons are pairwise, with condition 1 and 2 indicated

**1: Virgins with and without a germline**	
**Test: Impact of female germline in virgins**	
Condition 1	Condition 2
Virgin *tud/+* (no germline)	Virgin *tud/+* (germline)
**2: Virgin *vs.* 1- and 3- day post-mating**	
**Test: Impact of female germline and mating**	
Condition 1	Condition 2
Virgin *tud/+* (germline)	Mated *tud/+* (germline) female to Berlin male
Virgin *tud/+* (no germline)	Mated *tud/+* (no germline) female to Berlin male
**3: Virgin *vs.* 1- and 3- day post-mating**	
**Test: Impact of male germline and mating**	
Condition 1	Condition 2
Virgin Berlin	Mated Berlin female to *tud/+* (germline) male
Virgin Berlin	Mated Berlin female to *tud/+* (no germline) male

Given that the female mutants, strains, and time points examined here for gene expression changes have not been systematically examined for post-mating behavioral changes, we examine post-mating sleep, food preference for yeast- or sucrose-containing media, and female re-mating, and compare to previous studies (for comparisons see Table S1). We discover that both daytime and nighttime sleep are increased post-mating in *tud* progeny females without a germline, whereas nighttime sleep is decreased post-mating in control *tud* progeny females with a germline. This sleep result is distinct from previous studies that found daytime post-mating sleep decreased in all strains but *white* Berlin (Isaac *et al.* 2010; Garbe *et al.* 2016; Dove *et al.* 2017). We find that the female post-mating preference for yeast-containing media is independent of presence of eggs and receipt of sperm. A requirement for the female germline in the post-mating preference for yeast was not directly tested (Ribeiro and Dickson 2010; Vargas *et al.* 2010), nor was a requirement for sperm, just a role for sex-peptide (Ribeiro and Dickson 2010). For female re-mating, we find that the presence of sperm has an impact, but not the presence of eggs. Female re-mating is high when females are mated to males that do not transfer sperm, but not when females are infertile, due to lack of germline tissues at both one- and three-day(s) post-mating. This result is consistent with previous studies using other mutants that cause females to lack a germline (Chapman *et al.* 2003; Liu and Kubli 2003; Peng *et al.* 2005; Barnes *et al.* 2008).

It is clear that there are differences in gene expression and behavior due to strain/genotype. This prompted us to examine how genetic background influences female re-mating behavior, using two collections of wild-caught *Drosophila* strains (Mackay *et al.* 2012; Campo *et al.* 2013). A genome-wide association study (GWAS) identified several significant polymorphisms and indels in or near genes, including four genes in the Wnt signaling pathway and several genes with known nervous system expression.

## Materials and Methods

### Fly stocks and maintenance

The wild type Berlin strain was used for gene expression analyses. In the sleep analysis, both wild type Berlin and Canton-S (CS) are used. Animals without a germline and genetically identical control animals with a germline were produced from crosses using *tudor* (*tud*) females, a recessive, maternal-effect allele. Progeny from homozygous *tud* mutant mothers do not form pole cells; these progeny lack germline tissues, but the somatic tissues of the gonad are present (Boswell and Mahowald 1985; reviewed in Thomson and Lasko 2005). The genotype of experimental and control *tud* progeny were all the same genotype (*tud^1^*, *bw^1^*, *sp^1^/+*), but were produced using a different crossing scheme. Animals that lacked germline tissues were the progeny of *tud^1^*, *bw^1^*, *sp^1^* females (mothers are homozygous for *tud^1^*) and Berlin males; animals with germline tissues were the progeny of *tud^1^*, *bw^1^*, *sp^1^/SM1* females (mothers are heterozygous for *tud^1^*) and Berlin males.

Age-matched virgin and mated females were generated by collecting *tud^1^*, *bw^1^*, *sp^1^/+* virgin females (with and without a germline), Berlin virgin females, naïve *tud^1^*, *bw^1^*, *sp^1^/+* males (with and without a germline), and naïve Berlin males in groups of 11, 0-6 hr post-eclosion. All flies were aged for five days (for the three-day post-mated time-point) or seven days (for the one-day post-mated time-point), to ensure all female flies were eight-days old at the time of collection. *tud^1^*, *bw^1^*, *sp^1^/+* virgin females (with or without a germline) were mated with Berlin males, and Berlin virgin females were mated to *tud^1^*, *bw^1^*, *sp^1^/+* males (with or without a germline). Males and females were mated at a 1:1 male to female ratio, for 24 hr. We found 24 hr was a sufficient amount of time to ensure 100% of females were mated, as assayed by the presence of progeny (data not shown).

All flies were raised at 25° under 12:12 hr light-dark cycle and grown using standard cornmeal food media (33 L H_2_O, 237 g Agar, 825 g dried deactivated yeast, 1560 g cornmeal, 3300 g dextrose, 52.5 g Tegosept in 270 ml 95% ethanol and 60 ml Propionic acid).

### Library preparation

Flies were briefly anesthetized under CO_2_ and males were removed. Mated females were returned to their food vials and allowed to recover from CO_2_ treatment for eight hours (one-day post-mating time point) or aged for an additional 48 hr (three-day post-mating time point). Virgin Berlin and *tud^1^*, *bw^1^*, *sp^1^/+* females (with or without a germline) were collected shortly following eclosion and aged for eight days. All females were collected by rapidly tapping the flies into vials without anesthesia, immediately snap frozen in liquid nitrogen, and stored at -80°.

Adult heads were separated from bodies by mechanically tapping frozen cryovials on a hard surface. The heads were then sorted from other body parts on plastic cooled on dry ice, to keep tissues frozen. Approximately 100 heads per sample were immediately transferred to TRIzol (Invitrogen). Total RNA from heads was extracted using Trizol, and polyA mRNA was purified using MicroPoly(A) Purist columns (Ambion). All subsequent steps of the Illumina library preparation were performed as previously described (Masly *et al.* 2011). The libraries were sequenced from a single end, using an Illumina Genome Analyzer IIx sequencer, with 72 bases determined. There were three independent biological replicates for all conditions.

### RNA-sequencing read mapping

The Illumina reads were aligned to the *Drosophila* reference genome FB5.51 (FlyBase v5.51) using Bowtie 2, a Tophat alignment tool (version 2.0.8 Langmead *et al.* 2009). The count table was extracted from the Tophat files using easyRNAseq (version 3.0.2) and FPKM values were calculated using cufflinks (version 2.1.1, Delhomme *et al.* 2012; Trapnell *et al.* 2012). Statistical analyses to determine differential gene expression were performed for each pairwise comparison using the “tagwise” model of dispersion in the edgeR statistical package (version 3.0.2, Robinson *et al.* 2010). FDR correction was performed on all contrasts to correct for multiple testing and false positives (Benjamini and Hochberg 1995). Significant differences in gene expression were determined at an FDR corrected q-value < 0.05, only testing genes that passed a filter of FPKM >1 in all three replicates, in at least one condition, to filter out genes with low expression. The full table of results is provided (Table S2).

### Quality control and validation

A principal component analysis was performed on data for all genes that passed filter in at least one condition (9,352 genes), using the online tool iDEP.82 (http://bioinformatics.sdstate.edu/idep/, Figure S2, Ge *et al.* 2018). Correlation across replicates was performed using the JMP statistical software (JMP, Pro 13. SAS Institute Inc.), with the replicates showing high correlation. To determine the relatedness of biological replicates, we performed cross-correlation analysis for each experimental condition, using a Pearson’s Product-Moment correlation with a row-wise estimation (Figure S3). Correlation across replicates was *r* >0.9, for all conditions, with most having an *r* >0.97. Thus, differences in the numbers of genes with expression differences in the comparisons are not due to differences in variance across the replicates from any one condition.

Additionally, qRT-PCR was performed using independent head samples than those collected for RNA-sequencing. A set of genes were chosen based on the RNA-seq results, as significantly differentially expressed, with a fold-change >2 and FDR < 0.05 (Figure S4**).** These genes were *Diptericin B* (*DptB*), *Drosomycin* (*Drs*), *female-specific independent of transformer* (*fit*), *Metchnikowin* (*Mtk*), *target of brain insulin* (*tobi*), and *Vago*. Three biological replicates of approximately 40-50 heads were collected for each replicate, in each condition, and homogenized into 1mL of TRIzol (Invitrogen). RNA was extracted using TRIzol, followed by an on-column DNase Digestion using RNA Clean & Concentrator™ -25 columns (Zymo Research) with rDNase (Machary-Nagel). cDNA was made using SuperScript III Reverse Transcriptase (Invitrogen), and qPCR was performed using SYBR green PCR Master Mix (Applied Biosystems) on a QuantStudio Flex (Applied Biosystems). Primer sequences are provided in Table S3. The 2^−ΔΔCt^ method (Livak and Schmittgen 2001) and internal control gene Rp49 were used to calculate expression levels (Figure S4).

### Gene ontology and pathway analysis

Gene Ontology (GO) and Pathway analysis were performed through the Flymine portal v45.1, using a Benjamini-Hochberg correction with a *P*-value cut-off of <0.05 (Lyne *et al.* 2007). The full list of results is available in Table S4.

### Gene list overlap analysis

To examine the number of gene lists for which the same genes have differential expression, we used an Upset plot for visualization (Lex *et al.* 2014), which is conceptually similar to a Venn diagram. The Upset plot shows the number of genes in each list (horizontal bar graph) and the number of genes that overlap across the lists (vertical bar graph). Statistical analysis of the overlapping genes across all pairwise comparisons was performed using the R package ‘GeneOverlap’ (Shen 2019). Significance of gene list overlap is calculated using a Fisher’s exact test that considers the number of genes overlapping, and the total number of genes in the genome (17,294 genes). We used the Jaccard Index to determine the amount of similarity between two lists. For the Fisher’s exact test and Jaccard Index, we used the 16 gene lists that include genes that were either induced or repressed by mating from this study (Table 2), as well as the top 100 genes that were induced and repressed at one- and three-days post-mating identified in our previous study, using the Canton-S strain (Dalton *et al.* 2010).

**Table 2:** Numbers of differentially expressed genes in each pairwise comparison, with induced and repressed gene numbers indicated separately

Differentially expressed genes in female head tissues				
Comparison	**(1) Virgin females with and without a germline**			
	Repressed due to absence of a germline (*tud/+*) (higher in virgin females with a germline)		Induced due to absence of a germline (*tud/+*) (higher in virgin females without a germline)	
Virgin	69		83	

**16 gene lists from post-mating comparisons**				
	**(2) Females with and without a germline mated to males with a germline**			
	♀ with a germline (*tud/+*)		♀ without a germline (*tud/+*)	
	♂ Berlin		♂ Berlin	
	Induced	Repressed	Induced	Repressed
1-day post-mating	430	279	182	104
3-day post-mating	269	256	1093	640

	**(3) Females with a germline mated to males with and without a germline**			
	♀ Berlin		♀ Berlin	
	♂ with a germline (*tud/+)*		♂ without a germline (*tud/+*)	
	Induced	Repressed	Induced	Repressed
1-day post-mating	320	365	248	277
3-day post-mating	137	146	220	199

### Re-mating behavioral assays

The virgin female and male flies were collected in groups of 10, shortly after eclosion, and aged for 4-7 days. Females were then mated in a 1:1 male: female ratio for 24 hr. After 24 hr, the flies were briefly anesthetized, males were removed, and females were returned to their original vials. To determine if re-mating occurred, for the second mating we utilized males that have fluorescent sperm (*w;P{w+mC,dj-GFP.S}AS1/CyO*; referred to hereafter as *DJ-GFP*). The dissected internal reproductive tract of females used in this assay was visualized using a Leica MZFLIII fluorescence stereomicroscope to detect the presence of *DJ-GFP* marked sperm. For the one-day post-mating time point, *DJ-GFP* males were added to the vials immediately after the first set of males were removed. The *DJ-GFP* males were added in a 1:1 male: female ratio and allowed to mate for an additional 24 hr. For the three-day post-mating time point, females were aged for an additional 48 hr, and then *DJ-GFP* males were added in a 1:1 male: female ratio for 24 hr. Following this 24-hour mating period, flies were briefly anesthetized, and males were removed. Re-mating was scored based on the presence of GFP in the female reproductive tract within six hours of the males being removed. Additionally, virgin females were collected and aged as above, but only mated with the *DJ-GFP* males in a 1:1 male: female ratio, as a control. ANOVA and Tukey-HSD *post-hoc* tests were performed in JMP Pro 14.0.0 (see Table S5).

### Sleep behavioral assays

Virgin females were collected and aged for five days. On day five they were mated to males or retained as virgins. On day six males were removed and female flies were individually loaded into 5 × 65mm glass tubes (Trikinetics Inc.), plugged on one end with 5% sucrose and 1% agar dipped in paraffin wax to seal. The non-food end was sealed with parafilm, with small air holes. The vials were loaded into *Drosophila* activity monitors (TriKinetics Inc.) and placed in a 25° incubator in 12:12 light: dark. Each condition was run for six days. The data from the first day of activity was not considered, as flies were recovering from CO_2_ anesthesia. Activity was measured as the number of beam breaks and collected in one-minute bins. Data were analyzed using ShinyR-DAM (Cichewicz and Hirsh 2018). ShinyR-DAM uses a sliding five-minute window to determine sleep events, where a sleep event is defined as five continuous minutes with no movement. ShinyR-DAM provides the mean number of sleep events per individual fly, separately for lights-on and lights-off (Cichewicz and Hirsh 2018); this is the data used for sleep analyses presented (Table S5). ANOVA and Tukey-HSD *post-hoc* tests on data from ShinyR-DAM were performed in JMP Pro 14.0.0, where daytime and nighttime sleep were analyzed separately (Table S5).

### Food preference behavioral assay

Food preference was performed as previously described (Ribeiro and Dickson 2010). Virgin females and naïve male flies were collected and aged for five days. Five-day old females were placed on sucrose agar food (100mM sucrose and 0.75% agar) and females were either kept as virgins or mated for 24-hours on day six (for three-day post-mating time-point) or day seven (for 1-day post-mating time point). On day eight, all females were briefly anesthetized with CO_2_ and placed on Petri dishes spotted with red food (20mM sucrose, 0.5 mg/ml of the red dye amaranth, and 0.75% agar) and blue food (5% yeast, 0.125 mg/ml of the blue dye indigo carmine, and 0.75% agar). Petri dishes were placed in a dark, 25° incubator for three hours. Subsequently, flies were flash frozen to be scored at a later date. Flies were scored for red, blue, purple, or no color in their abdomens. Groups of flies are scored as preferring yeast if >50% of the flies had blue abdomens. The percent of groups that preferred yeast was calculated. Each condition was run on multiple plates over multiple days.

### Genome-wide association study of re-mating behavior in natural strains

The re-mating behavior analyses were performed on F_1_ progeny from P_0_ *w^1118^* males crossed with females from either the *Drosophila* Genetic Reference Panel strain collection (138 strains; DGRP; Mackay *et al.* 2012), or strains from Winters, CA (28 strains; Campo *et al.* 2013). Males used for the re-mating assay were *w^1118^* (first mating) and *DJ-GFP* (second mating).

F_1_ virgins were collected in vial groups of 11 females and aged for 3-6 days. An average of six vial groups were collected for each F_1_ genotype, for a total of 1,076 vial groups. F_1_ virgins were then mated to *w^1118^* for 24 hr in a 1:1 male: female ratio. Following the 24 hr, flies were briefly anesthetized with CO_2_ and females were placed back into their original vials and aged for 48 hr. *DJ-GFP* males were then introduced into the vials of females and allowed to mate for 24 hr. After 24 hr, flies were briefly anesthetized with CO_2_ and females were singly placed into individual vials where they laid eggs for 12-14 days. The F_2_ progeny were scored to assess F_1_ re-mating based on the F_2_ eye color; if F_1_ females mated with the *DJ-GFP* males, a proportion of the F_2_ progeny will have orange eyes.

Percent re-mating was calculated from vial groups where eight or more F_1_ females survived the assay and produced 15 or more F_2_ progeny, in order to ensure re-mating could be reasonably assessed. It was calculated by taking the number of F_1_ females that re-mated divided by the total number of females in that vial group. Afterward, percent re-mating from each vial group was sorted from lowest to highest percent re-mating and ascending ranks were assigned based on this sorting (1-1,076). Ranks from each replicate for a single genotype were averaged together for the averaged rank transformed value. GWAS was performed on the rank transformed data from the F_1_ progeny from 138 DGRP strains, using the web-based pipeline at dgrp2.gnets.ncsu.edu (Huang *et al.* 2014). The DGRP2 workflow reports *P*-values from both a simple regression and a mixed effects model for polymorphisms in the DGRP panel (Huang *et al.* 2014). Given that the behavioral data set was generated from F_1_ progeny from crosses between DGRP females and *w^1118^* males, significant associations from the GWAS are likely due to dominant polymorphisms/indels in DGRP strains, but could also be due to recessive alleles present in both the DGRP and *w^1118^* strains, with the DGRP polymorphisms identified here.

### Data availability

All raw and mapped read data are available through the gene omnibus database under accession number GSE90724. Supplemental material available at figshare: https://doi.org/10.25387/g3.11317307.

## Results

A goal of this study is to determine how mating and presence or absence of a germline (hereafter referred to as germline status) influences gene expression changes and behavior, in order to gain insight into the cross-tissue coordination of reproductive physiology, behavior and metabolism. In this study, we examine females with or without a germline that are either virgin, one-, or three-days post-mating. We also examine how receipt of sperm and/or seminal fluid impacts gene expression and behavior, by assaying females that are mated to males with or without a germline at one- and three-days post-mating.

### Overview of Gene Expression Analysis

To understand how reproduction and cross-tissue interactions influence gene expression, we assay the global transcriptional responses in adult head tissues of age-matched females. To generate male and female animals without germline tissues, we performed a cross with P_0_ females that are either homozygous or heterozygous for the maternal-effect allele of *tudor^1^* (*tud^1^*). The males in the P_0_ cross are Berlin males. Progeny from homozygous *tud*^*1*^ mutant mothers do not have germline tissues, while progeny from heterozygous *tud*^*1*^ mothers have germline tissues. Thus, same-sex tud^*1*^ progeny, with and without a germline, are the same genotype (*tud^1^*, bw^*1*^, *sp^1^/+*; hereafter *tud/+*; see Methods for more detail). We conduct three separate comparisons that control for genetic strain background, within each comparison (Table 1). First, we examine gene expression in virgins, with or without a germline (*tud/+*, Table 1, comparison 1). Next, we examine the post-mating gene expression response at one- and three-days post-mating, in *tud/+* females (with and without a germline) mated to Berlin males (Table 1, comparison 2). In the third set of comparisons, we examine the post-mating gene expression response in Berlin females mated to *tud/+* males (with and without a germline, Table 1, comparison 3). For each condition, Illumina libraries were generated for three independent biological replicates. Differential gene expression is determined at an FDR < 0.05 and fold-change is calculated to determine direction of change (Table S2).

### The germline impacts gene expression in virgin female head tissues

To understand how germline tissue influences gene expression in the adult head, we identify genes with expression changes that are due to presence of the germline in virgin females. We identify 152 significantly differentially expressed genes, with 83 genes with higher expression in virgin females without a germline and 69 genes with higher expression in virgin females with a germline (Table 2 and Table S6).

An analysis of the enriched pathways using Kyoto Encyclopedia of Genes and Genomes (KEGG) and Reactome for the 152 genes reveals that the germline impacts genes with known roles in metabolism in virgin females, as all six significant pathways are involved in metabolism. We also use Gene Ontology (GO) to determine if there is enrichment of genes that function in a biological process (hereafter referred to as gene set enrichment analysis). Gene set enrichment analysis for the 152 genes further confirms significant enrichment for genes with known metabolic functions (Table S4). Based on the FlyAtlas tissue gene expression data set (Chintapalli *et al.* 2007), the 152 genes are known to have high expression in the head and fat body in wild-type animals, but low expression in brain tissue, indicating signaling between the germline and head fat body may generate many of the expression differences in this comparison.

#### Female germline regulation of genes that function in metabolic homeostasis:

Examination of the induced and repressed gene lists separately shows that the germline alters the expression of genes that are known to respond to changes in nutrition status and/or insulin signaling (Table S4 for enriched pathways and GO terms). The absence of germline tissues results in higher expression of genes known to signal high dietary nutrients, whereas presence of germline tissues results in higher expression of genes known to signal reduced nutrient storage and increased metabolic breakdown (reviewed in Droujinine and Perrimon 2016).

For example, in virgin females without a germline higher expression of *ilps* is observed. *ilps* are known to be induced by food uptake (Table S6; *ilp2*; fold change (FC)= 1.8, *ilp3* FC= 4.5, and *ilp5* FC= 3.1). Higher expression of the gene that encodes the neuropeptide *CCHamide-2* (FC= 1.6) is also observed. *CCHamide-2* is known to be induced by dietary sugar and proteins (Table S6; reviewed in Droujinine and Perrimon 2016). There is also higher expression of genes that code for energy storage molecules, including *yolk protein 3* (FC= 1.3) and *larval serum protein 2* (FC= 3.4; Table S6). Additionally, *target of brain insulin* is induced (FC= 1.6; Table S6), which is known to be induced by a high protein – low sugar diet (Buch *et al.* 2008), as is *female-specific independent of transformer* (FC= 2.4; Table S6), which is known to be induced by high protein intake (Sun *et al.* 2017). The majority of named genes in the list of genes with higher expression in females without a germline are known to be involved in nutrient sensing and notably also include: 1) *adipokinetic hormone receptor* (FC= 1.3), which functions to antagonize insulin signaling to mobilize fat stores (reviewed in Lehmann 2018), 2) *Niemann-Pick type C-2g* (FC= 1.3) which functions in sterol homeostasis and steroid biosynthesis (Huang *et al.* 2007) and 3) *Lipid storage droplet-1* (FC= 1.4) and *Phosphoenolpyruvate carboxykinase* (FC= 1.5; Table S6) which function in lipid storage (Patel *et al.* 2005; Okamura *et al.* 2007).

On the other hand, in virgin females with a germline, higher expression of genes that are annotated to have functions in nutrient breakdown are observed. These genes include: 1) *ilp6* (FC= 0.7) and the peptide hormone *limostatin* (FC= 0.6), both of which are known to be induced by cessation of feeding (reviewed in Droujinine and Perrimon 2016); 2) *brummer* lipase (FC= 0.7) and *bubblegum* (FC= 0.6) which function in lipid metabolism (reviewed in Liu and Huang 2013); 3) *1,4-Alpha-Glucan Branching Enzyme* (FC= 0.6), a hydrolase involved in the synthesis of glycogen (Paik *et al.* 2012); and 4) *InR* (FC= 0.6), the sole receptor known to bind Ilps1-7 (Table S6; reviewed in Nässel *et al.* 2013). Taken together, the results suggest that the germline is a critical driver of gene expression changes that are known to impact how energy stores are utilized or maintained.

### The impact of the female or male germline on gene expression changes post-mating

As mating has previously been shown to alter gene expression in female head tissues, with different responses seen across time (Dalton *et al.* 2010), we next determine how the presence of female germline tissues, or receipt of sperm, influences gene expression changes at one- and three- days post-mating. We compare expression in virgin and mated females with and without a germline (females are *tud/+* and males they are mated to are Berlin). We also compare expression in virgin and mated Berlin females that were mated to males with and without a germline (males they are mated to are *tud/+*; see Table 1 comparisons 2 and 3). Here, expression in virgin females is the baseline, so genes are either induced (higher in mated females) or repressed (higher in virgin females) by mating. This allows us to understand how an environmental change (mating) impacts cross-tissue interactions in females and how this differs depending on germline status in males and females.

There are 16 gene lists total (bottom of Table 2), given that we assay two time points (one- and three- day post-mating), and the impact of the female germline and male germline, with eight lists of genes with induced expression and eight lists of genes with repressed expression (see Table 2). The total number of genes with changes in expression is highest in *tud/+* females without a germline, three-days post-mating (1,733 genes), and lowest in Berlin females with a germline, mated to *tud/+* males with a germline, one-day post-mating (283 genes; Table 2). The other lists have an average of 525 +/− 146 genes with changes in expression (Table 2).

There is a larger number of genes that change expression three-days post-mating in females without a germline, compared to females with a germline mated to either fertile males or males without a germline (Table 2). This suggests that some gene expression changes are due to an interaction of receiving sperm and seminal fluid proteins and the absence of eggs. The differences are not only due to lack of production of fertilized eggs after mating, as we would expect a similar response in females mated to males that do not produce sperm, nor was the response only due to receipt of seminal fluid proteins after mating, as these proteins were transferred during mating in all conditions assayed here.

#### KEGG and Reactome pathway analysis:

**Figure 1:**
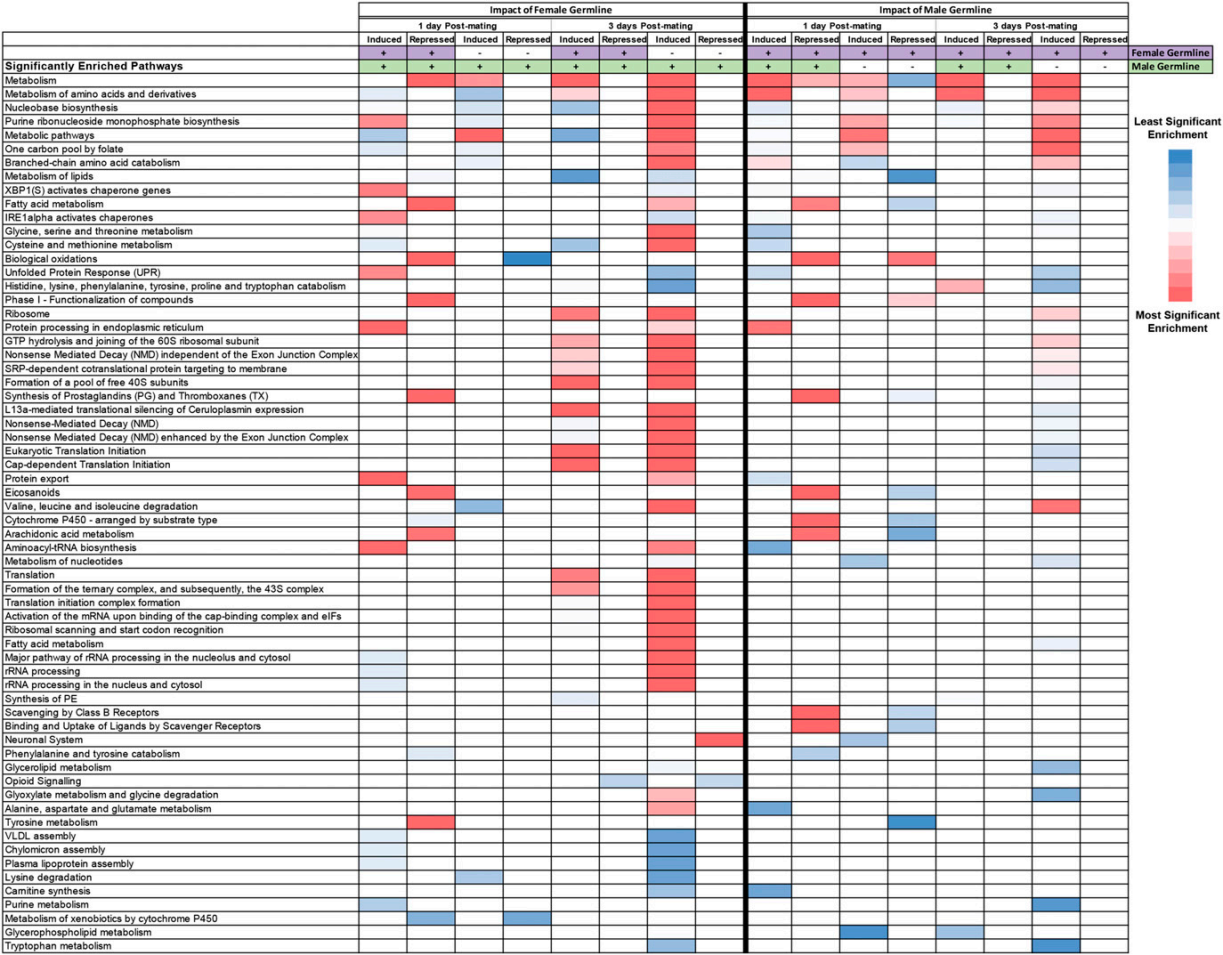
Summary of shared, enriched pathways. A comparison of enriched KEGG and Reactome pathways across 16 gene lists (Table 2). The significance of the *P*-values are indicated as a heat map with more significant values indicated in red (*P* = 2.38 × 10^−38^ for the most significant value), less significant values in blue (*P* = 0.05 for the least significant value) and median in white. *P*-values are listed in Table S4. The heat map was generated in Excel using a three-color scale across all conditions, with other values colored proportionally. The pathways are sorted with those at the top found in the most lists. Empty cells indicate that the pathway was not enriched in the list. The induced and repressed lists of genes from the comparisons that examine the impact of the female (left side) and male (right side) germline are shown. The female (purple) and male (green) germline status is indicated at the top, with (+ and color) indicating germline is present and (- and no color) indicating germline is absent. All pathways found in more than one list are presented; those that appeared in only one condition are in Table 3.

In order to determine if the gene expression changes in different conditions are due to genes with functions in the same pathways and processes, we first examine the enriched KEGG and Reactome pathways that are identified in the 16 gene lists (from Table 2). Genes annotated with functions in metabolic pathways were enriched in the majority of the comparisons we examined (Figure 1, Table 3, and Table S4), consistent with previous reports (Mcgraw *et al.* 2004; Dalton *et al.* 2010; Parisi *et al.* 2010).

**Table 3:** KEGG and Reactome Pathways that are uniquely enriched in the 16 lists of genes that are either induced or repressed by mating in female head tissues. If a list is not present that indicates that there were no unique enriched pathways identified. Females are either *tud/+* (with or without a germline) mated to Berlin males, or Berlin mated to males that are *tud/+* (with or without a germline)

Description of biological conditions for each list	Pathway	*p*-value	No. of Genes
Induced genes	Ribosome biogenesis in eukaryotes	5.60E-03	16
Female *tud/+* with germline			
1-day post-mating			
Repressed genes	Regulation of Insulin-like Growth Factor (IGF) transport and uptake by Insulin-like Growth Factor Binding Proteins (IGFBPs)	1.72E-02	7
Female *tud/+* with germline	Post-translational protein phosphorylation	1.72E-02	7
3-day post-mating	HuR (ELAVL1) binds and stabilizes mRNA	1.92E-02	3
Induced genes	Metabolism of carbohydrates	3.17E-11	50
	Pentose and glucuronate interconversions	1.27E-04	17
	Pentose phosphate pathway	1.49E-04	12
	Glycolysis / Gluconeogenesis	1.63E-04	19
	Glucose metabolism	1.69E-04	18
	Gluconeogenesis	2.28E-04	14
	Metabolism of RNA	2.67E-04	94
	Pentose phosphate pathway (hexose monophosphate shunt)	3.51E-04	9
	Metabolism of vitamins and cofactors	6.36E-04	28
	Amino acid synthesis and interconversion (transamination)	6.38E-04	10
	Propanoate metabolism	8.52E-04	11
Female *tud/+* with no germline	Metabolism of water-soluble vitamins and cofactors	1.34E-03	24
	Fructose and mannose metabolism	1.41E-03	13
	Triglyceride metabolism	1.42E-03	8
	Catabolism of glucuronate to xylulose-5-phosphate	2.65E-03	6
	Galactose metabolism	3.10E-03	12
	COPI-dependent Golgi-to-ER retrograde traffic	6.55E-03	11
	Pyruvate metabolism	9.49E-03	15
	Metabolism of folate and pterines	1.46E-02	9
	Metabolism of proteins	1.57E-02	152
	Metabolism of polyamines	1.64E-02	20
	beta-Alanine metabolism	2.07E-02	8
3-day post-mating	Arginine and proline metabolism	2.27E-02	15
	Plasma lipoprotein assembly, remodeling, and clearance	2.56E-02	10
	Amino sugar and nucleotide sugar metabolism	2.71E-02	14
	ABC-family proteins mediated transport	3.09E-02	12
	Starch and sucrose metabolism	3.09E-02	16
	Peroxisome	3.53E-02	17
	Ascorbate and aldarate metabolism	3.94E-02	10
	Fructose biosynthesis	3.95E-02	3
	Urea cycle	3.95E-02	3
	Ethanol oxidation	3.95E-02	3
	Triglyceride biosynthesis	4.67E-02	5
Repressed genes	Transmission across Chemical Synapses	9.94E-07	19
Female *tud/+* with no germline	Neurotransmitter release cycle	1.50E-04	9
3-day post-mating	Signaling by GPCR	3.03E-03	25
	Signal Transduction	3.65E-03	54
	Acetylcholine binding and downstream events	4.32E-03	5
	Glutamate Neurotransmitter Release Cycle	4.32E-03	5
	Postsynaptic nicotinic acetylcholine receptors	4.32E-03	5
	Activation of Nicotinic Acetylcholine Receptors	4.32E-03	5
	Phototransduction - fly	1.35E-02	7
	G alpha (q) signaling events	1.39E-02	6
	GPCR downstream signaling	1.43E-02	12
	Nephrin family interactions	1.47E-02	5
	Acetylcholine Neurotransmitter Release Cycle	1.47E-02	4
	Highly calcium permeable postsynaptic nicotinic acetylcholine receptors	1.78E-02	4
	DARPP-32 events	2.49E-02	3
	Neurotransmitter receptors and postsynaptic signal transmission	2.56E-02	9
	Collagen degradation	2.75E-02	4
	Reelin signaling pathway	2.75E-02	4
	Ca^2+^ pathway	2.78E-02	5
	Axon guidance	3.10E-02	20
	Developmental Biology	3.26E-02	23
	Extracellular matrix organization	3.26E-02	9
	PLC beta mediated events	3.71E-02	6
	Hemostasis	3.79E-02	23
	Basigin interactions	3.95E-02	5
	G-protein mediated events	4.11E-02	6
Induced genes	ECM-receptor interaction	2.91E-02	4
Berlin female mated to male *tud/+* with germline	Association of TriC/CCT with target proteins during biosynthesis	2.91E-02	4
1-day post-mating	Sulfur amino acid metabolism	3.59E-02	5
	Lysine catabolism	4.63E-02	4
Induced genes	Tryptophan catabolism	2.70E-02	4
Berlin female mated to male *tud/+* with no germline			
3-day post-mating			
Repressed genes	Smooth Muscle Contraction	2.14E-04	6
Berlin female mated to male *tud/+* with no germline	Muscle contraction	3.99E-03	6
3-day post-mating	FCERI mediated Ca+2 mobilization	2.61E-02	3
	CLEC7A (Dectin-1) induces NFAT activation	2.61E-02	3

Given that the enriched pathways we identify are shared across many of the different conditions we assay, we next examine the overlap. To do this, we display the enriched KEGG and Reactome pathways for all 16 gene lists (Figure 1), sorted by pathways that are shared across the most lists. The pathway ‘Metabolism’ is shared across the most lists (10/16 lists), with ‘Metabolism of amino acids and derivatives’ and ‘Nucleobase biosynthesis’ pathways enriched in all eights lists of genes induced post-mating. There are no pathways enriched in all eight lists of genes repressed post-mating. The pathway ‘Metabolism of lipids’ is enriched in gene lists from both repressed (3 lists) and induced (2 lists) genes. Overall, there are several pathways for metabolism and sub-categories for metabolism that are enriched across many of the induced and repressed lists.

We next analyzed the enriched KEGG and Reactome pathways that were unique to each condition (16 gene lists from Table 2), thus we only considered pathways that appeared in a single list (Table 3). Largely, these unique pathways are sub-categories of metabolic processes. However, the list of genes that are repressed by mating in females lacking a germline at three-days post-mating, is the only one with a large number of enriched neuronal-related pathways (Table 3). These pathways include: ‘Transmission across Chemical Synapses’, ‘Signal transduction’, ‘Axon guidance’, ‘Glutamate Neurotransmitter Release Cycle’, and ‘Acetylcholine Neurotransmitter Release Cycle’ (Table 3 and Table S4).

#### Female genotype impacts gene expression changes:

**Figure 2:**
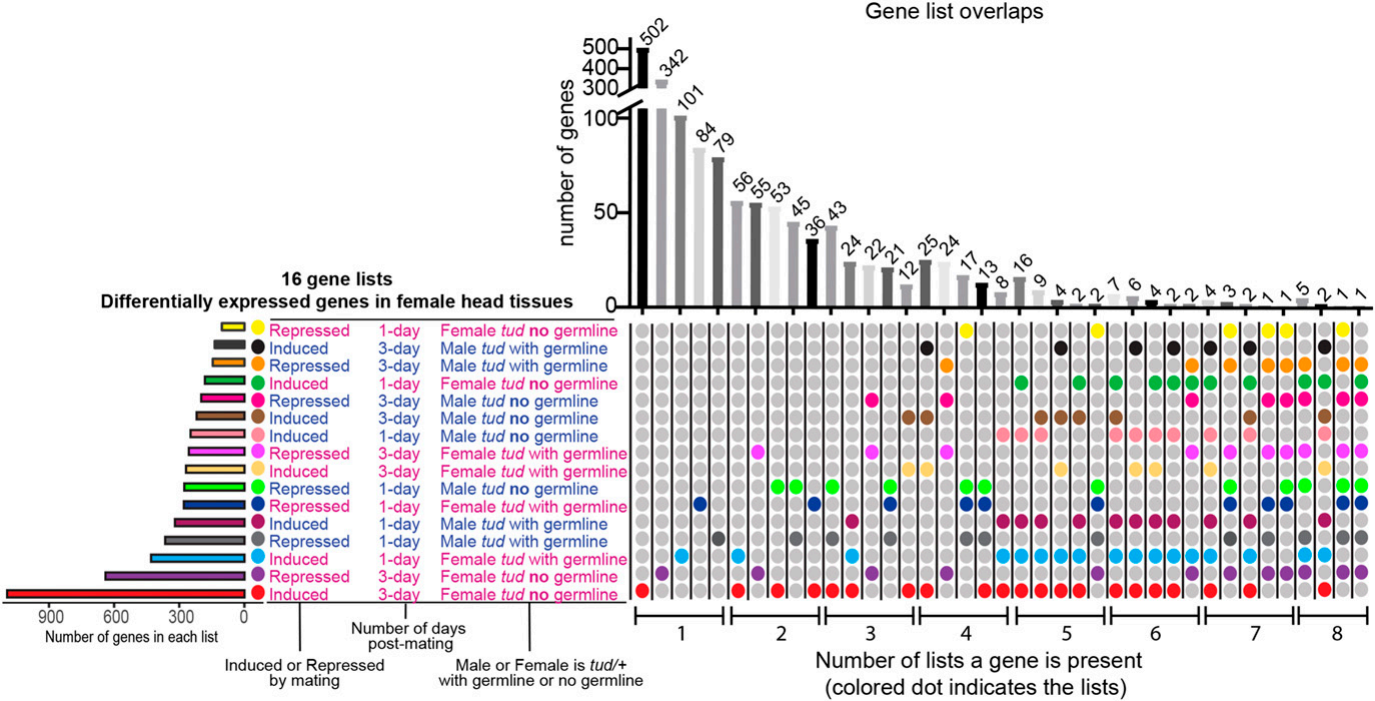
Overlap of differentially expressed genes. Comparison of the 16 lists of genes that were differentially expressed at one- and three-days post-mating, using an Upset plot, which is conceptually similar to a Venn diagram. The horizontal histogram at the left shows the number of genes in each of the 16 lists. The vertical histogram on the right shows the number of overlapping genes. The colored dots show the condition(s) where the gene(s) are present. The number of lists the gene is present within is indicated on the bottom, from left to right, going from one list to eight lists, with each category only showing the top five intersections.

Given that genes involved in metabolic pathways are enriched across all comparisons, we determine if this is due to the same or different genes changing expression in the 16 gene lists. We display the overlap of genes across the 16 gene lists (Table 2) using an ‘Upset plot’ (Figure 2, Lex *et al.* 2014), which is conceptually similar to a Venn diagram. We find there is limited amount of overlap of genes across the 16 gene lists. For example, the number of genes in common across any pairwise comparison includes only 36-56 genes, in the top five pairwise comparisons for overlapping gene lists. Furthermore, a maximum of five genes are shared across any eight gene lists (Figure 2). This demonstrates that expression of different genes were changing in the female head in the different conditions, and that the overlap of enriched pathways may largely be due to different genes or small numbers of genes.

We next determine the significance of the overlap of genes in each pairwise comparison for the 16 gene lists (from Table 2). We find that genes that are induced by mating in one condition, significantly overlap with the seven other lists of genes induced by mating (Figure S5 for results from Fisher’s exact test). The same result holds for genes that are repressed by mating (Figure S5). Therefore, the significant overlap from the Fisher’s exact test is due to a small number of overlapping genes, as is expected from the Upset plot analyses (Figure 2).

We find a similar result when we compare the genes with differential post-mating expression in females with a germline to those from our previous post-mating, gene expression dataset, in which we used the wild type Canton-S (CS) strain (Dalton *et al.* 2010). We compare the two lists of genes that changed post-mating in this study (females with a germline mated to males with a germline; *tud/+*, and Berlin females), to the top 100 genes by FDR rank from mated CS, from the previous study (Figure S5). At one- and three- days post-mating, we find a significant overlap among pairwise comparisons, due to a small number of genes overlapping (21 genes at one-day, and seven genes at three-days overlap across the three genotypes).

Gene expression differences due to genotype are also apparent in virgins used here. We compare *tud/+* females with a germline to virgin Berlin females. We find 428 differentially expressed genes. 181 genes are more highly expressed in the *tud/+* females and 247 genes more highly expressed in Berlin females (Table S6).

### The impact of the germline on sleep, food preference, and refractoriness post-mating

Given that each condition assayed had a different gene expression response (Table 2), we wondered if this results in behavioral differences. For example, the genes that changed expression three-days post-mating in females without a germline included an enrichment of GO terms related to sleep, including six terms related to the circadian sleep/wake cycle (Table S4). Here, we systematically characterize female post-mating behaviors in the genotypes and time points used in this study and compare to previous results (for comparisons see Table S1).

#### Sleep:

We examine differences in sleep post-mating, as to our knowledge, sleep has not been assayed in females lacking a germline (Table S1 for publication summary; for sleep statistical tests see Figure S6 and Table S5). Previous work has shown that mating results in decreased daytime sleep, across multiple strains, including CS, Oregon R, *iso^31^*, and *w^1118^*, but not *white* Berlin (see Table S1; Isaac *et al.* 2010; Garbe *et al.* 2016; Dove *et al.* 2017). In female strains with a germline (CS, Berlin and *tud/+*), we confirm that CS has a significant post-mating reduction in daytime sleep, whereas Berlin had a significant increase in daytime sleep (Figure S6A and B and Table S5). While others found no post-mating impact on nighttime sleep, we find a post-mating reduction in nighttime sleep (CS and *tud/+*; Figure S6A and B and Table S5).

**Figure 3:**
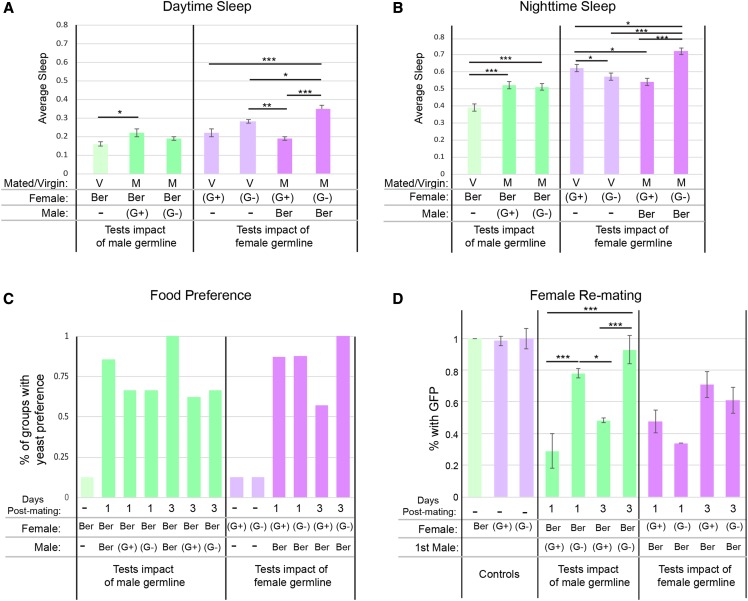
Effect of the germline on female reproductive behaviors. The female genotypes are Berlin (Ber, green) and *tud/+* (purple) with a germline (G+) and without (G-). The male genotypes are Berlin (Ber) and *tud/+* with a germline (G+) and without (G-). The virgin (V) and mated (M) status of females is indicated. The impact of mating and germline on daytime (A) and nighttime (B) sleep, averaged across days 2-6 post-mating is shown. For each fly, the mean sleep is determined by ShinyR-DAM. Each column shows the average of the mean sleep across all flies for each condition. Error bars show the standard error of the mean. (C) Preference for yeast-containing media *vs.* sugar-containing media post-mating. Bar graphs show the percent of groups that preferred yeast-containing media for each condition. The days post-mating (PM) is indicated (1 or 3 days). (D) Female re-mating was assayed. Average percent re-mating of vial replicates are plotted, with error bars showing the standard error of the mean. Statistical analyses were done using an ANOVA (see Table S5), followed by a Tukey HSD post-hoc test. The categorical values for the Tukey HSD results are indicated where *=*P* < 0.05, **=*P* < 0.005, and ***=*P* < 0.0005.

Previous studies showed that there is a sex-peptide-dependent, sperm-independent, post-mating decrease in daytime sleep that is sustained for multiple days (Isaac *et al.* 2010; Dove *et al.* 2017). For Berlin females, however, we find an increase in daytime sleep post-mating when Berlin females are mated to males with and without a germline (*tud/+*) (Figure 3A and Table S5), however the post-mating increase is only significant with males that had a germline. Additionally, we find a significant increase in nighttime sleep when Berlin females are mated to males with or without a germline (*tud/+*). Our results suggest that in Berlin females the post-mating sleep response may be impacted by receiving sperm.

To determine if the presence of the female germline impacts sleep, we examine post-mating sleep in female flies with and without a germline (*tud/+*). Presence of a germline in virgin comparisons (*tud/+*) does not impact daytime sleep, but there was a reduction in nighttime sleep in virgin females without a germline (*tud/+*) compared to controls with a germline (*tud/+*; Figure 3A and 3B).

Next we examine post-mating sleep in females with and without a germline. Post-mating, females without a germline (*tud/+*) show an increase in both daytime and nighttime sleep (Figure 3A and 3B). On the other hand, post-mated control females with a germline (*tud/+*), have a significant post-mating decrease in nighttime sleep (Figure 3B). Therefore, in conditions where we control for strain background of females and males (*tud/+* females and Berlin males), females with a germline have decreased nighttime sleep post-mating, whereas females lacking a germline have increased daytime and nighttime sleep post-mating. These changes in sleep are seen over multiple days post-mating (Figure S6C). Taken together, these data show a new type of cross-tissue interaction, with female fertility by mating interactions regulating the amount of sleep in females (for full statistical analyses of sleep see Table S5).

#### Food preference:

Previous work has shown that mated females have an increased preference for food containing yeast that is dependent on the sex-peptide pathway (Ribeiro and Dickson 2010; Vargas *et al.* 2010). It was also demonstrated that females that do not produce eggs (*ovoD* mutation) show a preference for yeast after yeast deprivation (Ribeiro and Dickson 2010). Here, we determine if the changes seen in post-mating food preference are affected by the female germline or the receipt of sperm, and whether that changes as time increases post-mating. We find that all females preferred yeast-containing media, over sucrose-containing media, at both one- and three- day(s) post-mating (Figure 3C). Therefore, we conclude that the change in preference for yeast-containing media is independent of fertility.

#### Refractoriness post-mating:

Finally, we investigate if the absence of a germline influences re-mating at both one- and three- days post-mating (Figure 3D). A previous study showed no differences in re-mating at one-day post-mating using *germ cell-less* females, a different maternal effect mutant that results in progeny without a germline (Jongens *et al.* 1992; Barnes *et al.* 2007). We also find that at both one- and three- day(s) post-mating, there are no significant differences in re-mating between *tud/+* females with and without a germline, with both showing re-mating around 30–40% at one-day post-mating, and 60–70% at three-days post-mating. Even though the genes that changed expression after mating are different, these differences do not appear to influence female re-mating. It is known that sex-peptide binds sperm and has an impact on both the short-term and long-term response of female re-mating (Chapman *et al.* 2003; Liu and Kubli 2003; Peng *et al.* 2005). Females mated to *tud/+* males with sperm have significantly lower percent re-mating than those females that were mated to *tud/+* males lacking sperm (Figure 3D), consistent with the observation that sperm is required for the decrease in female receptivity post-mating (Table S1; Kalb *et al.* 1993; Xue and Noll 2000).

### Percent re-mating is variable across a panel of inbred lines

**Figure 4:**
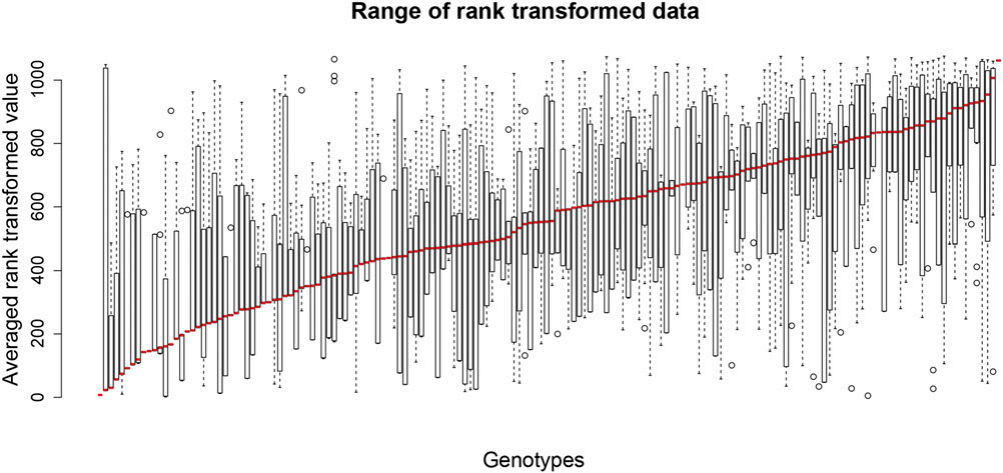
Genome Wide Association. (A) Phenotype plot in rank order. Boxplots illustrating the range of rank transformed re-mating (y-axis) for each strain (x-axis). Boxplots show quartiles via box and whiskers, and median with the bold black line. Outliers are single points outside of whiskers. Genotypes with percent re-mating and rank order are available in Table S7.

Previous studies showed differences in female fecundity and re-mating as a result of strain background, as well as the strain background of their mates (Fukui and Gromko 1989; Mcgraw *et al.* 2009; Chow *et al.* 2010; Chow *et al.* 2013; Delbare *et al.* 2017). Given the observed gene expression differences due to strain, we next determine if there is natural variation in female re-mating. We assay the F_1_ progeny derived from a cross between *w^1118^* males and females from either the *Drosophila* Genetic Reference Panel (DGRP; Mackay *et al.* 2012), or inbred lines derived from Winters, CA (Campo *et al.* 2013). The F_1_ progeny are from 166 different female P_0_ strains (138 DGRP and 28 Winters strains), with the rationale that heterozygosity is more akin to what is found in the wild. We observed variation in re-mating across the F_1_ progeny from the two panels of inbred lines (Figure 4, Table S7), with a similar range of percent re-mating between the two populations. The percent re-mating was 0–90% in DGRP lines and 0–87.5% in Winters lines (*P* = 0.1357, Student’s *t*-test).

Rank transforming the data resulted in satisfying the assumption of normality for the genome-wide association study (GWAS) model (Figure S7, Table S7). GWAS was performed using data from F_1_ progeny from the 138 DGRP strains. We use the web-based pipeline DGRP2, to identify associations due to polymorphisms in this population (Huang *et al.* 2014). This analysis identified significantly associated polymorphisms across the genome (top five are *P* = 4.6–9.8 × 10^−7^; the next ten are *P* = 2.2–3.7 × 10^−6^), including single nucleotide polymorphisms and indels. The top 100 significantly associated polymorphisms are in/near 59 unique, annotated genes (top 100 have *P* = 4.6x10^−7^–6.2 × 10^−5^, Table S8).

Examination of where these 59 genes have significantly high expression identifies the adult brain (29 genes), larval central nervous system (24 genes), and ventral nerve cord (25 genes), as the tissues with largest number of these genes with enriched expression (using the Flymine portal to examine Flyatlas data; Lyne *et al.* 2007; Robinson *et al.* 2013). Among the 59 genes, four are located in/near genes that are annotated to be involved in the Wnt signaling pathway (*Axin*, *Carrier of wingless*, *nemo*, and *wingless*). *Carrier of wingless* and *nemo* are in the top 20 most significant associations (Table 4). Though a specific role for Wnt signaling in female mating and re-mating has not previously been identified, it is a pathway that directs cell fate and physiology (reviewed in Nusse and Varmus 2012). Notably, the biological process ‘cell-to-cell signaling by Wnt’ (GO:0198738) is enriched in the list of genes that are changed by mating in *tud/+* females lacking a germline at three-days post-mating, suggesting that this signal-transduction pathway is important for the female long-term, post-mating response.

**Table 4:** Top 20 GWAS Associations. DGRP IDs where no gene was in region: 3R_21980124_SNP, 2L_19316857_SNP, 2L_19316859_INS, 2L_19316854_INS, 3L_14143779_DEL, 2R_19225235_DEL

DGRP2 ID	Gene Annotation	Biological Process	Molecular Function	Single Mixed P value
X_21373247_SNP	CR45082	—	—	3.85E-07
X_21373572_SNP	CR45082	—	—	5.53E-07
X_21373578_SNP	CR45082	—	—	5.53E-07
3R_18903893_SNP	Cow	Regulation of Wnt signaling pathway	Wnt-protein binding	8.71E-07
3R_18903892_SNP	Cow	Regulation of Wnt signaling pathway	Wnt-protein binding	1.82E-06
2L_6262204_INS	Ddr	Protein phosphorylation	Protein kinase activity	2.10E-06
2L_21290182_SNP	Mondo	Regulation of glucose metabolic process	Transcription factor binding	2.10E-06
3L_8019127_SNP	nmo	Negative regulation of Wnt signaling pathway	Protein kinase activity	2.82E-06
3L_3307167_INS	ZnT63C	Cellular zinc ion homeostasis	Cation transmembrane transporter activity	3.01E-06
3L_10336246_INS	CR46006	—	—	4.82E-06
3L_18127530_SNP	in 5′ region of Cyp312a1	Oxidation-reduction process	Heme-binding	5.70E-06
3R_13324673_SNP	Dscam3	Homophilic cell adhesion via plasma membrane adhesion molecules	Identical protein binding	5.98E-06
X_15992071_SNP	CG42354 and CG42353	—	—	8.13E-06
3L_4921078_SNP	in 3′ region of Rh50	Ammonium transmembrane transport	Ammonium transmembrane transporter activity	9.14E-06

Next we determine the specificity of our GWAS gene hits for female re-mating by comparing to other GWAS studies. We find that 25/59 genes were also identified in a study examining variation in *Drosophila* olfactory responses (significant overlap of gene lists is *P* <4.05 × 10-4 using the Flymine portal; Arya *et al.* 2010), suggesting that re-mating may have an olfactory component. There were no other GWAS publications found in the Flymine portal that had a significant number of genes that overlapped with our list of 59 genes. We also looked for overlap with several GWAS studies that examine behavior and find at most 5 overlapping genes between our study and others (Durham *et al.* 2014; Ivanov *et al.* 2015; Morozova *et al.* 2015; Nelson *et al.* 2016; Garlapow *et al.* 2017; Jehrke *et al.* 2018; Harbison *et al.* 2019), suggesting that the hits we find are fairly specific for female re-mating. Further functional studies will be important to understand the roles of these genes in female behavior.

## Discussion

*Drosophila* is a premier model system for studying cross-tissue interactions, given that *Drosophila* have organ systems that are similar to those found in mammals and the gene pathways that mediate cross-tissue interactions have evolutionary conservation (reviewed in Rajan and Perrimon 2011; Droujinine and Perrimon 2016). It is clear that signaling molecules that act at a distance coordinate female reproduction, egg production, nutrient homeostasis and behavior through changes in gene expression (reviewed in Rajan and Perrimon 2011; Droujinine and Perrimon 2013; Droujinine and Perrimon 2016). Here, we investigated the impact of 1) egg production in virgins, 2) female mating when she is sterile, and 3) female mating when the male is sterile, on gene expression changes in the adult female head. We also investigated how reproductive differences and strain differences impact a set of female post-mating behaviors.

In virgins, the presence of the germline changed expression of genes with known functions in nutrient homeostasis pathways, with females lacking a germline having increased expression of genes that are known to signal high dietary nutrients, and females with a germline having expression profiles consistent with reduction of nutrient storage and metabolic breakdown. It is unclear if these nutrient/energy signaling pathways are changed to stimulate germ cell production, or if the changes in expression are a result of larger nutrient reserves, or some combination. While females that are not producing eggs likely have more energy stores, previous studies showed that insulin levels directly control female germline stem cell division (Ikeya *et al.* 2002; Lafever and Drummond-Barbosa 2005; Hsu and Drummond-Barbosa 2009).

We also found that the presence/absence of a female germline altered expression of immune related genes, in virgins (Table S6). Previous studies showed that a post-mating induction of genes involved in the immune response requires a germline (Mcgraw *et al.* 2004; Mcgraw *et al.* 2008; Short *et al.* 2012; Short and Lazzaro 2013). Building on this, we show that the germline-dependent change in expression of immune-related genes occurs even in the absence of mating.

Interestingly, there were also changes in neurotransmitter-related genes in virgins due to absence of a germline (Table S6). Notably, some of these genes have previously been implicated in female reproductive behaviors. For example, *pale*, which encodes for the rate limiting enzyme in the synthesis of dopamine, was increased in virgin females lacking a germline (FC = 1.3), and dopamine is important in regulating female receptivity (Neckameyer 1998). On the other hand, *Neuropeptide-like precursor 3*, whose expression decreases post-mating (Mcgraw *et al.* 2008; Dalton *et al.* 2010), was also decreased in virgins lacking a germline (FC = 0.7). Taken together these results suggest that both mating and the female germline are important regulators of expression of neurotransmitter-related genes in adult head tissues.

For all the post-mating gene expression conditions examined, very few genes had expression changes in multiple, post-mating conditions assayed here (Figure 2). However, the genes with expression changes were enriched with those that function in metabolic pathways (Figure 1). Therefore, long-term, post-mating, gene expression changes in metabolic pathway genes do not require production of fertilized eggs, or receipt of sperm. A common aspect of the female mating conditions in this study is receipt of male Acps that are transferred in the male seminal fluid (reviewed in Ravi Ram and Wolfner 2007; Avila *et al.* 2011), suggesting that their transfer, or the sensory aspect of mating (Shao *et al.* 2019), has a sustained impact on expression of genes involved in metabolism in female head tissues.

A previous study that examined female, whole-animal, post-mating gene expression changes in response to sperm (no Acps), Acps (no sperm) and mating (no Acps, no sperm), also found that transfer of sperm, male seminal fluid proteins or mating caused unique changes in gene expression, or differences in the magnitude of gene expression changes (Mcgraw *et al.* 2004). Taken together, the many different studies examining post-mating gene expression changes in females show that the post-mating time point, tissue assayed, and if the male transfers sperm or Acps have a large impact on gene expression changes that are detected (Lawniczak and Begun 2004; Mcgraw *et al.* 2004; Mack *et al.* 2006; Kapelnikov *et al.* 2008; Mcgraw *et al.* 2008; Innocenti and Morrow 2009; Mcgraw *et al.* 2009; Dalton *et al.* 2010; Parisi *et al.* 2010; Gioti *et al.* 2012; Short and Lazzaro 2013; Fear *et al.* 2016; Delbare *et al.* 2017).

On the other hand, females without a germline, three-days post-mating was the only post-mating condition that had an enrichment of several ‘neuronal’ and ‘behavioral’ biological process genes with expression changes. Genes involved in GABA synthesis (*Gad1*, FC = 0.7) and transport of glutamate (*VGlut*, FC = 0.7) were both repressed by mating at three-days post-mating. Glutamatergic and GABAergic neurons are widespread in the *Drosophila* nervous system and have been associated with sleep and olfactory sensing (Liu and Wilson 2013; Zimmerman *et al.* 2017). *Adar* (FC = 0.7), which is also included in this list of genes, has also been shown to effect sleep by repressing glutamatergic signaling (Robinson *et al.* 2016). Additional genes that encode for receptors for the neurotransmitters acetylcholine, dopamine, and octopamine had decreased expression in females lacking a germline at three-days post-mating. Previous studies have implicated acetylcholine as a mediator of learning and memory, visual perception, and olfaction (Shinomiya *et al.* 2014; Barnstedt *et al.* 2016), which are all important for female post-mating behaviors. Furthermore, both octopamine and dopamine have been shown to induce female post-mating behaviors, namely egg-laying, sperm storage and female receptivity to mating (Neckameyer 1998; Monastirioti 2003; Avila *et al.* 2012; Rubinstein and Wolfner 2013; Heifetz *et al.* 2014; Rezával *et al.* 2014).

We note that our study may not detect expression changes for genes with low expression in the nervous system. For example, it is clear that *doublesex*-, *fruitless*-, and *pickpocket*-expressing populations of neurons underlie female mating behaviors (Häsemeyer *et al.* 2009; Yang *et al.* 2009; Rideout *et al.* 2010; Rezával *et al.* 2012), but we did not identify these genes here, suggesting additional cell-type and single-cell gene expression experiments would provide new insights into additional genes critical for behavioral changes.

We determined if reproductive status also caused different behavioral responses post-mating. All post-mating female conditions assayed changed their food preference to yeast-containing media, instead of sucrose-containing media. The females did differ in their re-mating response, with females mated to males lacking sperm showing the highest percent re-mating, whereas females that lack a germline re-mate at similar levels to their control with a germline, as was also previously shown using different strains to generate females that lack a germline (Barnes *et al.* 2007). When we examine post-mating sleep changes, females without a germline show significantly increased sleep during the day and night, whereas control females with a germline have significantly reduced sleep during the night.

For sleep, it has previously been shown that artificially activating glutamatergic neurons in the brain leads to increased wakefulness, therefore inhibiting these neurons could result in increased sleep (Zimmerman *et al.* 2017). We found genes that function in glutamate neurotransmitter release are repressed post-mating, in females that lack a germline, which could contribute to increased sleep. Furthermore, it is known that nutrient depletion reduces sleep and increases activity (Lee and Park 2004; Keene *et al.* 2010; Yang *et al.* 2015; Yu *et al.* 2016). Given that sterile females likely have more stored nutrients, this could also contribute to increased sleep. Similarly, mating is known to increase nutritional demands (Ribeiro and Dickson 2010; Vargas *et al.* 2010; Walker *et al.* 2015), which could explain the decrease in sleep seen post-mating in some strains when females have a germline. Along these lines, the observed strain differences we found in sleep post-mating may be due to strain differences in metabolism.

Our behavioral studies on F_1_ heterozygotes made from crosses from 166 wild-caught isogenic strains, demonstrated that there is a large range of re-mating behavior. A previous study showed that there is natural variation in sperm competition in females (Chow *et al.* 2013). This suggests that in wild populations, females may have different strategies in terms of mating, re-mating, and behaviors that maintain homeostasis, like sleep and feeding. We found four Wnt signaling pathway genes are associated with variation in re-mating. Though the Wnt signaling pathway has not yet been implicated in the regulation of female post-mating behavior, Wnt signaling is necessary for female fertility in mammals (Boyer *et al.* 2010), and for long-term memory formation in *Drosophila* (Tan *et al.* 2013). Given that the GWAS will identify genes that could have an impact during development, and on any tissue, it is not unexpected that we would find different genes than identified in our gene expression analyses.

Our examination of natural variation had additional similarities to the study examining sperm competition (Chow *et al.* 2013). We found that F_1_ progeny made from DGRP Ral313 had low re-mating, with only ∼7% of females re-mating. Ral313 never re-mated among 39 tested females from the DGRP collection that were used to examine sperm competition (Chow *et al.* 2013). Another similarity is that 15 of the 33 top associated polymorphisms are in/near neurological genes, three of which encode for ion channels (Chow *et al.* 2013). These three ion channel genes all had significantly higher expression in wild-type females mated to males lacking a germline, at one-day post-mating, making these genes better validated candidates for further functional and evolutionary studies.

Decreased production of eggs and sperm naturally occurs during aging (reviewed in Pizzari *et al.* 2008; Miller *et al.* 2014). Thus, our results together with those from other laboratories point to ways that the changes in the female environment (mated *vs.* unmated), reproductive senescence in both males and females, along with other changes, such as nutrition, can differentially influence gene expression through cross-tissue interactions (Pletcher *et al.* 2002; Gershman *et al.* 2007; Dalton *et al.* 2010; Parisi *et al.* 2010; Doroszuk *et al.* 2012; Gioti *et al.* 2012; Whitaker *et al.* 2014; Zhou *et al.* 2014). These rippling effects on gene expression ultimately impact physiological and behavioral phenotypes, and are also influenced by natural variation in the population. While we only examined gene expression in head tissues in females of different reproductive status, impacts on gene expression in other tissues and other phenotypes are likely to be widespread. Understanding cross-tissue interactions during *Drosophila* reproduction provides a powerful, systems-level model to study gene-by-environment interactions, the functions of genes during different stages of the life span, and how natural variation influences these functions.
